# Editorial: Exercise, diet, cytokines and obesity, volume II

**DOI:** 10.3389/fendo.2026.1774325

**Published:** 2026-01-21

**Authors:** Junhao Huang, Xu Yan, Bing Shen, Liwei Xie, Chia-Hua Kuo

**Affiliations:** 1 Guangdong Provincial Key Laboratory of Physical Activity and Health Promotion, Guangzhou Sport University, Guangzhou, Guangdong, China; 2 Dr. Neher’s Biophysics Laboratory for Innovative Drug Discovery, State Key Laboratory of Quality Research in Chinese Medicine, Macau University of Science and Technology, Macao, Macao SAR, China; 3 Institute for Health and Sport, Victoria University, Melbourne, VIC, Australia; 4 Guangdong Institute of Microbiology, Guangdong Academy of Science, Guangzhou, China; 5 Laboratory of Exercise Biochemistry, Education University of Hong Kong, Hong Kong, Hong Kong SAR, China

**Keywords:** cytokines, diet, exercise, inflammation, obesity

Obesity remains one of the most critical global health crises of the modern era (1). For a long time, obesity has been considered as a simple equation of calories in versus calories out (2) Meanwhile, the traditional paradigm of obesity management often narrowly focused on weight loss. Currently, we understand that obesity is not only related to excess adiposity but also is a systemic chronic low-grade inflammation condition that contributes to its severe comorbidities (3). This inflammatory environment, driven by a dysregulated secretion of cytokines and hepatokines from adipose tissue and the liver, creates a vicious cycle of metabolic dysfunction and insulin resistance with increased risk for cardiovascular disease, type 2 diabetes, and sarcopenia (4). The articles in this Research Topic demonstrated how lifestyle interventions, particularly exercise and nutrition, work not merely by creating a calorie deficit, but by modulating this inflammatory environment, which offers a more powerful and sustainable framework for obesity management.

The narrative review by Sánchez et al. elucidates the critical role of pro-inflammatory cytokines such as TNF-α, IL-1β, and the dual-role of IL-6 in obesity’s pathophysiology. Importantly, it points out that exercise and nutrition are not just simple calorie-balancing tools, but are potent modulators of this immune-metabolic cross-talk. Acting as a “myokine therapy,” exercise stimulates the release of anti-inflammatory molecules from muscle, while anti-inflammatory dietary patterns can attenuate the production of pro-inflammatory cytokines.

The pilot randomized controlled trial from Yu et al. reported the feasibility and multifaceted health benefits of a 12-week online interactive Baduanjin exercise in adults with overweight and obesity. While the reduction in BMI was modest, the significant improvements in waist circumference, fatigue, anxiety, depression, and sleep quality were demonstrated. Reducing waist circumference, a marker of visceral fat, could directly inhibit the secrection of pro-inflammatory adipokines. Furthermore, by ameliorating psychological distress and sleep disorders, which are both causes and consequences of inflammation, could break the psycho-physiological loops that sustain obesity condition. This study highlights the benefits of exercise extend far beyond BMI.

The study from Liao et al. directly compared the effects of aquatic and land-based high-intensity interval training (HIIT) on selected bio- and physiological variables among obese adolescents. Both modalities effectively improved body composition, blood pressure, and low-density lipoprotein cholesterol in 4 weeks, highlighting HIIT as a time-efficient strategy. Furthermore, the superior improvements in lean body mass, waist circumference, waist-to-hip ratio, vital capacity, and total energy consumption in the aquatic HIIT group are particularly noteworthy. For individuals with obesity, who often face barriers like joint pain and mobility limitations, aquatic exercise provides a unique, low-impact environment that may enhance tolerability, safety, and enjoyment, which are critical for adherence.

Delving deeper into the molecular mechanisms, the research by Ataeinosrat et al. reported the effects of different exercise regimens on the modulation of hepatokines, which are liver-derived proteins. The finding revealed that 12 weeks of HIIT and Tabata training were more effective than moderate-intensity continuous training (MICT) in reducing levels of fetuin-B, FGF-21, FGL-1, and selenoprotein P. These hepatokines are implicated in promoting insulin resistance, hepatic steatosis, and inflammation. Their downregulation following high-intensity exercise offers a direct biochemical explanation for the metabolic improvements after such exercise interventions.

In conclusion, the aim of obesity management is not just for weight loss, but for the restoration of systemic health and resilience. However, it requires us to comprehensively understand the underlying mechanisms related to immune-metabolic cross-talk between muscle, liver, and adipose tissue. The evidence presented in the present Research Topic supports that a synergistic combination of regular and accessible exercise programs and anti-inflammatory dietary patterns is a powerful non-pharmacological therapy for obesity management.
